# Life satisfaction and longitudinal changes in physical activity, diabetes and obesity among patients with cardiovascular diseases

**DOI:** 10.1186/s12889-017-4925-0

**Published:** 2017-12-02

**Authors:** Michèle Baumann, Anastase Tchicaya, Nathalie Lorentz, Etienne Le Bihan

**Affiliations:** 1 Research Unit INSIDE, Institute Health & Behaviour, University of Luxembourg, Belval Campus, L-4366 Esch-sur-Alzette, Luxembourg; 2 Luxembourg Institute of Socio-Economic Research (LISER), Esch-sur-Alzette, Luxembourg

**Keywords:** Life satisfaction, Cardiovascular risk factors, Longitudinal changes in adherence, Diabetes, Obesity, Physical activity, Cohort

## Abstract

**Background:**

Patients with cardiovascular disease who underwent coronary angiography at the *National Institute of Cardiac Surgery and Cardiological Intervention* (INCCI) in Luxembourg were surveyed for cardiovascular risk factors (CVRF) (hypertension, hypercholesterolemia, diabetes, obesity, physical inactivity, tobacco consumption). In 2013/14, their life satisfaction (LS) was also assessed. Our aim was to analyse the relationships between LS on one hand and longitudinal changes in CVRF between 2008/09 and 2013/14 and socioeconomic factors on the other.

**Methods:**

1289 patients completed a self-administered questionnaire. Life Satisfaction, originally recorded on a 1 to 10 scale of complete satisfaction was dichotomized into two groups: ≤ 7 *and.* >7. We then performed logistic multiple regressions. The event on which the probability was modelled, was LS > 7. Data were adjusted on age, sex and income. Longitudinal changes in CVRF were assessed by their presence or absence in 2008/09 and 2013/14 (categories: ‘no-no’; ‘no-yes’; ‘yes-no’; ‘yes-yes’).

**Results:**

Physical activity in 2008/09 and 2013/14 was associated with a lower LS (OR = 0.469). The same pattern was observed for obesity and physical inactivity: lower LS was related to the presence of these risks (yes-yes; no-yes) in 2013/14 (mean OR for obesity and physical inactivity in 2013/14: 0.587 and 0.485 respectively), whereas their presence or absence in 2008/09 was not related to LS. Finally, patients who suffered from diabetes in 2008 were more likely to experience a decline in LS, particularly if their diabetes was less severe in 2013/14 (OR = 0.462).

**Conclusions:**

The lowest LS was observed when obesity or physical inactivity was present in 2013/14, newly or otherwise. The same trend was seen in diabetes among patients who had it in 2008/9, but were less severely affected in 2013/14. In secondary prevention, CVD-related upheavals could be minimised if professionals and patients became ‘*Partners in Healthcare*’ to better adhere to healthy lifestyles, as well as to reduce CVRF, and thereby enhance LS.

## Background

In spite of a considerable decline during recent decades, cardiovascular diseases remain the leading cause of death in most OECD countries, accounting for nearly a third of all deaths in 2013 [1]. As a result of advances in treatment and intervention strategies, more people are surviving coronary heart disease-related events [2] and living with the associated burden of chronic disease, which could impact on satisfaction with life. Life satisfaction represents the perceived degree of discrepancy between individual aspirations and achievements, and contentment [3, 4]. Satisfaction with one’s own life has been the focus of many studies in the past decade both within and across countries, during a single time period and over time (i.e OCDE and Eurofound reports) [5–7]. In the same context, research using various medical diagnostic groups highlighted that life satisfaction in patients with chronic diseases varied widely between different subgroups, for example, life satisfaction in patients with cardiac infarction was higher than in those with diabetes type I. The satisfaction with life of patients was associated with angina, but not with myocardial infarction [8]. A global conclusion is that the objective severity of illness does not correlate very highly with LS [9]. The associations of co-morbidities such as diabetes, hypertension and obesity, and high levels of stress, caused by the disease itself, physical limitation and risk of death, represent a vicious circle that intensifies disease progression [10], which could represent an aggravating factor for the decline in life satisfaction. Elucidation of life satisfaction and its determinants in secondary prevention remains necessary; the follow--up of cohorts is particularly under-documented.

In reality, the prospect of a new decline in cardiovascular disease is likely to be compromised by the rise of cardiovascular risk factors such as obesity and diabetes [1], and the fact that they cause long-term disabilities [11], and affect life satisfaction [8, 12]. Secondary prevention has become an essential aspect of care of the patient with coronary heart disease [11]. A recent study in secondary prevention observed that the satisfaction with life of patients with cardiovascular risk factors (i.e obesity, diabetes, hypercholesterolemia and physical inactivity) was more likely to be lower [13]. A limited number of studies concerning follow-up of cohorts examine the evolution of the changes over time of cardiovascular risk factors. A beneficial or deleterious impact on satisfaction among patients who are in a period of life heavily influenced by work would show that life satisfaction and adaptation to various life events can differ by age and sex [14].

After a cardiac infarction, patients must reorganise their daily lives, adapt to preventive behaviour, adjust to their new lifestyles and available income, as well as rethink their plans for the future. It is well established that early management of cardiovascular risk factors through longitudinal lifestyle changes, secondary prevention and/or therapeutic intervention leads to a marked reduction in mortality and morbidity [15, 16]. Non-adherence to preventive measures is a powerful confounder of evidence-based practice and can affect daily patient management, resulting in inappropriate therapeutic escalation; it increases the risk of adverse cardiac events, including mortality [17]. It is recognized that patients with hypertension or hyperlipidaemia tend to take less than half of their prescribed medications [18]. Indeed, cardiac rehabilitation and secondary prevention programmes have developed from focusing on exercise alone to becoming multidisciplinary and encompassing baseline patient assessments, nutritional counselling, risk factor management (i.e., lipids, hypertension, weight, diabetes, and smoking), psychosocial and vocational counselling, and physical activity advice and exercise training, in addition to the appropriate use of cardioprotective drugs [11].

Adherence to medical advice, therapeutic programs and secondary preventive measures modifies risk factors, and thereby may impacts on LS. Longitudinal investigations may be of interest to identify psychosocial, cognitive and material factors that contribute to adherence to treatment/medication and/or the adoption of healthy behaviour, and which intervene on the gap between the intention or motivation of the patients to adjust their lifestyle and the feeling that they are able or unable to change their behaviour such as suggested by the empirical approach of Godin [19] and the conceptual approach of Sheeran [20]. Comprehension of these interactions has become an important criterion of research where greater knowledge may be useful in improving procedures used to follow up CVD patients. Indeed, a better understanding of the variability over time of the changes of CVRF in secondary prevention may help individuals and policy makers make better decisions that increase or maintain patients’ life satisfaction. In this context, the national of Luxembourg eHealth agency (National Agency for Shared Information in Health) has given patients the right to receive all available information about their health status and treatment options in order to help them make informed choices. This applies mostly to patients with chronic diseases such as cardiovascular diseases. This reform supports keeping personalized medicine high on the political agenda, in line with the European cross-border directive of 2011 [21]. Our aim in this study is to analyse the relationships between longitudinal changes between2008/9 and 2013/14, in cardiovascular risk factors (hypertension, hypercholesterolemia, diabetes, obesity, physical inactivity, and tobacco consumption) and socioeconomic factors on one hand, and their satisfaction with life in 2013/14 on the other hand.

## Methods

### Study design

This study is part of the Monitoring and Dynamics of Health Status through the Risk Factors for Cardiovascular Disease (MDYNRFC) project, which aimed to assess the evolution of health status and cardiovascular risk behaviour. Five years after a coronary angiography undergone at the *National Institute of Cardiac Surgery and Cardiological Intervention* (INCCI) in Luxembourg in 2008/09, the patients of this cohort were contacted in 2013/14 for a follow-up study [22].

### Ethical aspects

This study was approved by the National Research Ethics Committee and the National Commission for Data Protection of Luxembourg. Before data collection, patients were asked to complete and to sign and return in a stamped envelope provided a written informed consent form after being informed of the research objectives.

### Study population and sample

Data derived from a follow-up study of 4391 patients admitted for a coronary angiography in INCCI from 1 January 2008 to 31 December 2009. Patients were contacted again 5 years later (August 2013–April 2014) by a letter that contained information about the aims of the survey and a questionnaire.

In total, 1837 questionnaires were accessed (including 548 deaths), representing a response rate of 42% compared to the patient population of the base year 2008/9. Excluding deaths, information on only 1289 patients could be used in the follow-up study.

### Organisation of the data collection and instrument

In 2013/14, the follow-up survey was conducted by mail with a self-administered questionnaire in one of three languages: Portuguese, French and German. Luxembourg is multilingual and very culturally diverse (more than 170 different nationalities). The three language versions of the questionnaire were translated and back-translated then proofread by native-speaking professional translators. Due to the lack of information concerning LS at the baseline, this study was cross-sectional for LS, but longitudinal for changes in CVRF.

### Measurement in 2013/14 alone

- Life satisfaction LS (dependent variable) was assessed as in the European survey [12]. All respondents self-rated their level of LS from 1 to 10 (completely satisfied) on a scale with one item concerning ‘*satisfaction with your life*’.

The few studies looking at LS and coronary artery disease measure LS using a self-reported tool. Some studies measured LS with one item about the global LS as ‘General LS’ [12, 23] and others used various life domains in an LS index that averaged domain satisfactions into a single composite (i.e. evaluated specific areas of life such as employment, family, sex life, leisure activities and standard of living) [6, 8, 9, 23]. However, these composites correlate strongly with global assessments of LS and exhibit stability across time. A meta-analysis demonstrated that the asymptote for multiple item measures was the same as for single-item measures [24].

- Socioeconomic data gathered were: age, sex, marital status, nationality, educational level, and professional status, occupation (retired) and total annual income in euros.

### Longitudinal data in 2008/9 and in 2013/14

*-* CVRF: diabetes, hypertension, hypercholesterolemia, weight and height were self-reported. Based on the International Obesity Task Force convened by the World Health Organization, a subject with a Body Mass Index (BMI) ≥ 30.0 kg/m2 is defined as obese, 25.0–29.9 kg/m^2^ is considered overweight and <25.0 kg/m^2^ normal. Tobacco consumption and physical inactivity were also recorded*.*

### Statistical analyses

Depending on their nature, variables were described using means, standard deviations or percentages. As the distribution of LS was highly skewed to the right, we did not use the usual linear multiple regression because the assumption of residuals normality would be violated. Therefore, LS was dichotomized as low (≤7) or high >7; the sample is thus equitably distributed. The European indicator of LS was 7/10 for EU-27 [12].

We then performed logistic multiple regressions of the dichotomized LS on socio-demographic variables and on longitudinal CVRF modifications. The event, in which the probability was modelled, was LS greater than 7. Adjustments were made for age, sex and income. Longitudinal changes in CVRF were assessed by their presence or absence in 2008 and 2013 (four categories: ‘no – no’; ‘no-yes’; ‘yes-no’; ‘yes-yes’). The links between LS and other variables had been expressed as odds ratios (OR), an OR higher or lower than 1 indicating a beneficial or deleterious link, respectively, between the probability of having an LS higher than 7 and the modality of the explanatory variable relative to the reference category. All analyses were performed with SAS 9.3 statistical software (SAS Institute Inc., USA).

## Results

### Participation rate and description of the sample (Table 1)

We received 1289 completed questionnaires, giving a response rate of 35.5% (1289 / 3635). Overall, respondents’ profiles differed from those of non-respondents, except for age, hypercholesterolemia and BMI. Respondents were more likely than non-respondents to have Luxembourg nationality, to be married or living with a partner, to be educated, to be retired, and to practice a physical activity. Respondents were less likely to be smokers, and to have diabetes and hypertension.

**Table 1:** Comparison of socio-demographic characteristics (reported in 2008) of respondents and non-respondents (excluding invalid addresses)

	Respondents	Non-respondents	P^§^
	*N* = 1289	*N* = 2346	
Age (years)	64.1 (11.2)	64.5 (12.6)	0.3924
Gender			
Male	70.7	64.6	0.0002
Female	29.3	35.4	
Nationality			
Luxembourgish	74.8	70.2	0.0040
Other	25.2	29.8	
Marital status			
Married	75.5	66.8	<.0001
Single/divorced/widowed	24.5	32.2	
Level of education			
Primary	44.5	56.2	<.0001
Secondary	40.0	31.3	
Superior	15.5	12.5	
Professional status			
Manual worker	28.8	35.7	<.0001
Employee	36.3	29.4	
Executive	8.9	5.8	
Other	26.0	29.1	
Occupation			
Retired	70.3	64.2	0.0004
Other	29.7	35.8	
Hypertension			
Yes	60.1	69.0	<.0001
No	39.9	31.0	
Hypercholesterolemia			
Yes	60.3	62.6	0.1879
No	39.7	37.4	
Diabetes			
Yes	23.5	29.4	0.0002
No	76.5	70.6	
BMI			
Normal	25.2	26.4	0.2260
Overweight	44.4	41.4	
Obese	30.4	32.2	
Physical activity			
Yes	68.0	51.9	<.0001
No	32.0	48.1	
Tobacco consumption			
Yes	17.1	25.9	<.0001
No	82.9	74.1	

^§^Significant *p*-value: *p < 0.05; **p < 0.01; ***p < 0.001

Means (s.d.) or %

### Socioeconomic characteristics (left-hand column of Table 2)

Approximately half of the sample had an LS higher than 7. Mean age was 69.2 years and there were more men than women, more Luxembourgish than other nationalities and more living in a couple. The majority had reached secondary education and were manual workers or employees, but about 4/5 of all patients were retired and 2/3 had a total annual income above the ‘high to middle’ figure.

**Table 2:** Life satisfaction and sociodemographic characteristics reported in 2013

			Life satisfaction^a^			
		Mean (s.d.) or %	OR	SE	p^b^	
Life satisfaction	≤7	46.6				
	>7	53.4				
Age		69.2 (11.1)	1.004	0.005	0.445	
Sex	Women	28.7	1.447	0.191	0.005	**
	Men	71.4	1			
Nationality	Luxembourgish	77.5	1.106	0.158	0.482	
Marital status	Living in a couple	74.0	1.197	0.168	0.200	
Level of education	Primary	31.9	0.750	0.150	0.292	
	Secondary	52.1	0.765	0.138		
	Higher	16.0	1			
Professional status	Manual worker	24.6	0.923	0.187	0.863	
	Employee	37.5	0.856	0.165		
	Executive	24.0	0.929	0.195		
	Other	13.9	1			
Occupation	Retired	78.1	1.012	0.190	0.950	
Total annual income (euros)	<18,000 (‘low’)	4.7	0.444	0.136	<.0001	***
	18,000–35,999 (‘low to middle’)	29.2	0.457	0.071		
	36,000–53,999 (‘high to middle’)	33.7	0.729	0.110		
	54,000 + (‘high’)	32.4	1			

^a^LS had been dichotomized according to ≤7 vs > 7. Regression model adjusted with age, sex and income

Other variables were introduced one at a time

^b^Likelihood ratio tests

Significant *p*-value: *p < 0.05; **p < 0.01; ***p < 0.001

Simple description (left-hand column) and logistic regression model adjusted for age, sex and income (right-hand columns)

### Socioeconomic characteristics and associations with LS (right-hand columns of Table 2)

Men were more likely than women to have an LS > 7 (OR = 1.447). With respect to the highest income bracket (≥54, 000 euros / year), ORs associated with lower income were all below 1, increasing with the income. We noticed no association between LS and age, nationality, living in a couple, educational level, professional status or retirement.

### Longitudinal changes in CVRF between 2008 and 2013 (left-hand column of Table 3)

CVRF remained mostly stable between 2008 and 2013 (yes 2008 – yes 2013 or no 2008 - no 2013). The main differences were among those who declared (yes) in 2008 and (no) in 2013 to hypertension (26.4%) and/or hypercholesterolemia (26.0%). Among the whole group of patients, 13.1% stopped participating in physical activity, whereas 14.2% took it up. The smoking cessation rate was 8.8%, close to the percentage of patients who continued to smoke (8.5%).

**Table 3:** Life satisfaction in 2013 and longitudinal changes in cardiovascular risk factors in 2008 and in 2013

		Description	Life satisfaction^a^			
		%	OR	SE	p^b^	
*CVRF*						
Hypertension	Yes 2008 - Yes 2013	37.8	0.782	0.119	0.090	
	Yes 2008 - No 2013	26.4	0.692	0.115		
	No 2008 - Yes 2013	5.6	0.616	0.179		
	No 2008 - No 2013	30.3	1			
Hypercholesterolemia	Yes 2008 - Yes 2013	38.2	0.813	0.126	0.170	
	Yes 2008 - No 2013	26.0	0.844	0.144		
	No 2008 - Yes 2013	10.2	0.604	0.141		
	No 2008 - No 2013	25.6	1			
Diabetes	Yes 2008 - Yes 2013	24.3	0.676	0.105	0.015	*
	Yes 2008 - No 2013	3.7	0.462	0.167		
	No 2008 - Yes 2013	6.4	0.736	0.199		
	No 2008 - No 2013	65.7	1			
Obesity	Yes 2008 - Yes 2013	23.8	0.652	0.092	0.002	**
	Yes 2008 - No 2013	7.0	0.903	0.213		
	No 2008 - Yes 2013	8.1	0.528	0.118		
	No 2008 - No 2013	61.2	1			
Physical activity	No 2008 - No 2013	19.5	0.469	0.083	<.0001	***
	No 2008 - Yes 2013	14.2	0.744	0.144		
	Yes 2008 - No 2013	13.1	0.501	0.102		
	Yes 2008 - Yes 2013	53.2	1			
Tobacco consumption	Yes 2008 - Yes 2013	8.4	0.946	0.202	0.554	
	Yes 2008 - No 2013	8.8	1.236	0.266		
	No 2008 - Yes 2013	1.6	0.649	0.303		
	No 2008 - No 2013	81.2	1			

^a^LS had been dichotomized according to ≤7 vs > 7. Regression model adjusted with age, sex and income

Other variables were introduced one by one

^b^Likelihood Ratio tests

Significant p-value: *p < 0.05; **p < 0.01; ***p < 0.001

Simple description (left-hand column) and logistic regression model adjusted with age, sex and income (right-hand columns)

### Longitudinal changes in CVRF and their relationship to LS (right-hand columns of Table 3)

Longitudinal changes in diabetes, obesity and physical activity were significantly linked to low LS (7 or less). More precisely, the presence of one of these CVRF in 2008 and in 2013 was associated with a lower LS, particularly for physical inactivity (OR = 0.469). The greatest modifications in obesity and physical inactivity on LS show a similar pattern: associations with LS were related to the presence of one of these risks (yes-yes; no-yes) in 2013, whereas they were least in 2008. In contrast, when one of these risks disappeared in 2013, associations with LS were more positive, but not entirely so (LS > 7) (OR for obesity and physical inactivity 0.903 and 0.744, respectively). However, this trend was not observed in diabetes. Patients who suffered from diabetes in 2008 were more likely to experience a lower LS, especially if they did not report it in 2013 (OR = 0.462).

Comparison of the OR of patients who did not have the hypertension or hypercholesterolemia risk factor in 2013 with those who always had it, indicates that ORs are close (hypertension: 0.692 vs. 0.782, hypercholesterolemia: 0.844 vs. 0.813); these risk factors are not associated with an improvement in LS. Similarly, the evolution of tobacco consumption does not seem to be associated with LS. However, it should be noticed that the percentages of non-smokers in 2008 and 2013 are particularly high, resulting in high standard errors of the ORs of other categories and a lower power of this test compared to other risk factors.

## Discussion

Our research examined the associations between longitudinal changes in cardiovascular risk factors and satisfaction with life among cardiovascular disease patients, 5 years after coronary angiography. With regard to our major findings, first, a lower LS was observed when obesity or physical inactivity was already apparent or became so in 2013/14. Second, the same trend was not seen in patients with diabetes that was recorded in 2008/9, but only when it appeared in 2013/14. Third, the decrease in LS was lower when obesity and physical inactivity disappeared in 2013–2014. We will develop these findings point by point.

One of our most interesting findings was that the lowest LS was more likely when failure to exercise was declared in 2013/14. Although our results did not investigate gender differences, we propose to underline the conclusions of a study [25] which examined the change in physical activity-mediated gender-related satisfaction with life over a 2-year period. It showed that higher physical activity levels impact on satisfaction with life positively, but observed that males were engaged in more physical activity than were females. A systematic review of the impact of lifestyle interventions in the secondary prevention of coronary heart disease follow-up of 3 months [26] confirmed that the overall results for modifiable risk factors suggested improvements in dietary and exercise outcomes. Hypotheses could be proposed to better understand the link between non adhesion to physical activity and satisfaction with life. Some of the patients probably had little motivation to adopt healthy behaviours or were not able to change [20], when they declared their inactivity 5 years after their event; they perceived their satisfaction with life negatively. In another study [27], data sets predicting behaviours in the health domain (smoking, applying universal precautions, exercising) were examined. Patients whose intentions were more aligned with the norms of the society were more likely to report healthy behaviours than were participants whose intentions differed [27]. Other suggestions that concerned reducing TV or computer time as well as targeting other healthy behaviours (eg. increasing physical activity levels, improving dietary intake) might prove useful [28]. In contrast, the more patients waste time by not becoming active, the more cardiovascular risk factors appear. In a Luxembourgish study [28], higher weekday sitting time was related to poorer cardiovascular health; time spent watching television was inversely associated with this score on both workdays and days off. Physical activity has been associated with improved physical fitness, social functioning, self-esteem, body image, mood, stress response; and a decreased risk of heart disease, and diabetes [29]. A longitudinal study of older people demonstrated that more time watching television was associated with incident central obesity after adjustment for covariables including physical activity, but not with total obesity when measured by BMI [30].

A second finding showed the same low trend on life satisfaction as previously when the risk of obesity was present in 2008/9 and in 2013/14, and when obesity appeared in 2013/14. In light of the previous hypothesis, and such proposals as those by Sheeran [20], we would suggest that during the 5 years after coronary angiography, these patients probably had most difficulty adapting their lifestyle and their life satisfaction had been perceived negatively because their intentions were questionable concerning medical advice and secondary prevention. Some authors [31] tried to account for the gap between knowledge of risk factors and change in unhealthy behaviour. Their conclusions indicated that intention is a moderate predictor of behaviour and that the gap between intention and behaviour is caused by high intenders not taking action. It is also necessary to recall that with long-term cardiovascular disabilities, social and familial repercussions on daily life, restrictions in routine, leisure and work activities, and socioeconomic impact, a proportion of patients go through a phase of depression and anxiety [32].

Another finding concerned longitudinal modifications in diabetes. The relationship with life satisfaction was deleterious, whereas lifestyle was more beneficial when obesity and physical inactivity disappeared in 2013/14. Patients who adjusted their lifestyles during the 5 years following a coronary angiography, are, we suppose, probably motivated, but are likely to have changed their behaviour against their will. They perceived adopting healthy behaviours as unpleasant, which may impact negatively on their life satisfaction. Effectively, patients who follow the strict regime prescribed and hence go from diabetes in 2008/9, to at least less severe diabetes in 2013/14, experienced a difficult period during which they maintained unpleasant lifestyle behaviours. In addition, eating carbohydrate-rich food is part of a ‘good life’ [1], but also a form of dependency. To abandon that and to limit ones diet and adopt physical activity could be devastating. Adherence to therapeutic advice and adjustment in lifestyle, including healthy eating and reducing the sugar intake in the diet, can reduce cardiovascular risk factors, and modify the satisfaction with life. For example, patients who must adopt a behaviour that is perceived as unpleasant, such as a diabetic regime, are affected emotionally because the gap between what they currently do and what they must do is likely to precipitate psychological difficulties. A better comprehension of the cognitive aspects of cardiovascular disease in relation to the affective aspects [33, 34] makes clear that what happens in the mind has to be taken into account if we want to understand how adapted lifestyles are adopted and how the ability to adhere to medication can be developed. Knowledge of the emotional impact of adherence may be useful for policy interventions aimed at improving the health and daily life of patients. Non-adherence is complex and remains difficult to define. In addition, the ability of providers to accurately identify at-risk patients is limited. Improved screening tools are needed to detect them and thereby facilitate appropriate targeting of interventions. Given the rapidly expanding global population with coronary heart disease and emerging clinical and cost-benefits of adherence, addressing non-adherence to prescribed therapies is a top priority [35].

Lastly, two other findings are worthy of a brief mention; the facts that being a woman and/or having a low income affect satisfaction with life. Firstly, in accord with this finding, in the presence of coronary artery disease, female-patients with co-morbidities such as diabetes, hypertension and obesity, presented greater impairment of their wellbeing. The unfavorable outcomes months after percutaneous coronary intervention were associated with female sex, a previous event, or procedure failure [36]. Another example, the rates of obesity and overweight vary according to educational level and socioeconomic status, and these disparities are significant among women, but less clear among men [37]. Secondly, the lower the income of study patients, the lower the life satisfaction. Lack of income would be greater already due to facing lifestyle disadvantages, and the consequences of CVD-related behaviours would be greater when they are confronted with new socioeconomic disadvantages like wages for domestic helpers. A study over time found that life satisfaction is lower when all the years of poverty are together rather than split up into periods in and out of poverty. This ties in with the idea that individuals may have the resources (financial or otherwise) to deal with relatively short-term poverty but not when it lasts too long [14].

Furthermore, our results showed that hypertension and hypercholesterolemia were not discriminant factors of the life satisfaction between patients with or without these risk factors. In other words, the link between the constraints related to treatments and to lifestyle changes of these risk factors, and the life satisfaction would be less strong than those associated to diabetes or obesity. Regarding the lack of a link between the evolution of tobacco consumption and LS, we can conclude that stopping smoking in a situation of medical stress can be experienced with difficulty.

### Strengths and limitations

#### The strength of the survey

resides in the population sample; the small size of the country made it possible to organize data collection at a national level. Our participation rate of 35.5% is similar to that of two previous studies (32.2% vs. 28%) in Luxembourg [28, 38]. Such study protocols are rare because they are very expensive and difficult to organise. Some patients died, moved to institutions, changed their residential arrangements (for example to live with a son or daughter), or failed to respond.

#### Some limitations might be indicated

Based on monitoring data from a cohort of patients five years after coronary angiography, a potential risk exists due to the current composition of the respondents of the follow-up group compared to the non-respondents regarding certain individual characteristics, except age, and certain cardiovascular risk factors: diabetes, hypertension, BMI, tobacco consumption and physical inactivity. Such limitations are inherent to most follow-up studies, particularly with cohorts of patients (mean age 64 years). Another limitation is the fact that life satisfaction was not measured at baseline at the time of examination of coronary angiography. It was not possible to measure satisfaction with life change during the study interval as well as the evolution of changes in cardiovascular risk factors. As a result, the relationship between life satisfaction and the evolution of risk factors cannot be interpreted as causality. [The follow-up surveys have not considered at the beginning of the design of the study to collect information concerning the presence of other pathologies, comorbidity, clinical and mental health variables (such as depression, anxiety and psychological distress), which could explain, at least to some extent, the differences obtained, for example in comorbid depression [39]. Therefore for further research, a control could be required of the consequence of the emotional state of CVD patients to be careful about the interpretation of the links between each cardiovascular risk factor and the satisfaction with life.

### Practical and clinical implications

A hypothesis can be suggested to explain the minimal positive relationship with life satisfaction observed in 2013/14 when obesity and physical inactivity disappeared. Attempts to understand behaviour among patients who took part in a secondary preventive program and medical follow-up should explore further the importance of internalized norms and self-expectations in the development of motivation and the ability to adopt a given behaviour [27].

Intensive diet and physical activity interventions have been found to reduce CVD risk, but are resource intensive. The American Heart Association recently recommended motivational interviewing (MI) as an effective low-intensity intervention to promote health-related outcomes such as weight loss. In a UK primary-care setting, low-intensity MI counselling was effective in bringing about long-term changes in some, but not all, health-related outcomes (walking, cholesterol levels) associated with CVD risk. Intervention was particularly effective for patients with elevated levels of CVD risk factors at baseline [40].

Taken together, other aspects probably intervened in longitudinal modifications in diabetes, obesity and/or physical activity, and associations with patient LS. A previous study [41] looked at the impact of the patient’s communication with the medical practitioner on adherence to preventive behaviours. It showed that good doctor-patient communication was related to nutrition, particularly increased consumption of fresh fruits and vegetables. Accurate perception of CVRF by both patient and medical practitioner is essential for CV protection. The aim of instructing patients is to encourage them to make informed decisions about how to change their lifestyle.

Cardiovascular secondary prevention with nurse-based telephone follow-up was more effective than usual care in improving low-density lipoprotein cholesterol levels 12 months after discharge for patients with diabetes mellitus or chronic kidney disease [42]. In the same time, to improve anxiety outcomes of patients following myocardial infarction, a telephone service can help patients and their family caregivers to adapt. Delivered by trained health and social professionals and comprising up to 10 telephone-delivered ‘health coaching’ sessions (ProActive Heart) [32] and combined with psychologically-specific treatment, this programme could impact on anxiety of greater intensity in a clinically meaningful way. Furthermore, therapeutic coaching provides reassurance about secondary treatment effects, improves adherence to prescriptions, and provides information about medical-social services [43]. In addition, for some patients, internet mobile application can help increase healthy behaviour in nutrition, such as vegetable consumption [44].

## Conclusion

Our research highlights the fact that information about the determinants of the satisfaction with life help explain certain difficulties encountered in secondary prevention. Maximising the comprehension of the potential of adherence, patients and professionals must discuss their respective roles as *‘Partners in Healthcare’* [45] and suggest that CVD-related upheavals can be minimised if they provide empowerment in diabetes, obesity and/or physical activity. Our findings call for further research into secondary prevention concerning the relation over time of socioeconomic conditions and the changes of cardiovascular risk factor related behaviours with patients’ life satisfaction. There may also be a need to evaluate the impact of psychosocial and material barriers or facilitators in the implementation of actions that would develop patients’ capability to adhere to medical advice and to adopt healthy lifestyles, as well as to reduce cardiovascular risk factors and enhance satisfaction with life.

## References

[1] OECD. Cardiovascular disease and diabetes: policies for better health and quality of care, Editions. OECD; 2015. https://doi.org/10.1787/9789264233010-en. Accessed 23 Nov 2017.

[2] Leon AS, Franklin BA, Costa F, Balady GJ, Berra KA, Stewart KJ (2005). Cardiac rehabilitation and secondary prevention of coronary heart disease: an american heart association scientific statement from the council on clinical cardiology (subcommittee on exercise, cardiac rehabilitation, and prevention) and the council on nutrition, physical activity, and metabolism (subcommittee on physical activity), in collaboration with the American association of cardiovascular and pulmonary rehabilitation. Circulation.

[3] Diener E, Biswas-Diener R (2002). Will money increase subjective well-being? A literature review and guide to needed research. Soc Ind Res.

[4] Diener E, Ng W, Harter J, Arora R (2010). Wealth and happiness across the world: material prosperity predicts life evaluation, whereas psychosocial prosperity predicts positive feeling. J Pers Soc Psychol.

[5] OECD. Guidelines on Measuring Subjective Well-being, OECD Publishing. 2013. ISBN 978–92–79-28315-4 (PDF). https://doi.org/10.1787/9789264191655-en. Accessed 22 Nov 2017. 24600748

[6] Schimmack U, Krause P, Wagner GG, Schupp J (2010). Stability and change of well being: an experimentally enhanced latent state-trait-error analysis. Soc Indic Res.

[7] D’Ambrosio C. An Overview of Intertemporal Measures of Individual Well-Being: Can They Explain Life Satisfaction Better? In Tachibanaki, Toshiaki (Ed.) Advances in Happiness Research. A Comparative Perspective*.* Part of the Creative Economy book series (CRE). 2016; 20(1): 39–54. ISBN 978-4-431-55753-1.

[8] Boehm JK, Peterson C, Kivimaki M, Kubzansky LD (2011). Heart health when life is satisfying: evidence from the Whitehall II cohort study. Eur Heart J.

[9] Henrich G, Herschbach P (2000). Questions on life satisfaction (FLZ^M^). Eur J Psychol Assess.

[10] Legrand VMG, Serruys PW, Unger F, van Hout BA, Vrolix MCM, Fransen GMP (2004). (on behalf of the arterial revascularization therapy study (ARTS)). Three-year outcome after coronary stenting versus bypass surgery for the treatment of multivessel disease. Circulation.

[11] Nichols M, Townsend N, Scarborough P, Rayner M (2013). Cardiovascular disease in Europe: epidemiological update. Eur Heart J.

[12] Eurofound. Quality of life in Europe: subjective well-being. Luxembourg: Publications Office of the European Union, 2013. ISBN 978–92–8971–120-3.

[13] Baumann M, Tchicaya A, Vanderpool K, Lorentz N, Le Bihan E (2015). Life satisfaction, cardiovascular risk factors, unhealthy behaviours and socioeconomic inequality, 5 years after coronary angiography. BMC Public Health.

[14] Clark A, D'Ambrosio C, Ghislandi S. Poverty Profiles and Well-Being: Panel Evidence from Germany. In Thesia I. Garner, Kathleen S. Short (ed.) Measurement of Poverty, Deprivation, and Economic Mobility (Book Series: Research on Economic Inequality). Emerald Group Publishing Limited, 2015;23:1–22. ISBN: 978–1–78560-387-7 eISBN: 978–1–78560-386-0. Accessed 22 Nov 2017.

[15] Institute for Health Metrics and Evaluation. The Global Burden of Disease Study 2010. USA, Seattle. [www.healthmetricsandevaluation.org].

[16] WHO (2013). A global brief on hypertension. Silent killer, global public health crisis.

[17] Kolandaivelu K, Leiden BB, O'Gara PT, Bhatt DL. Non-adherence to cardiovascular medications. Eur Heart J*.* 2014; 7, 35(46):3267-3276. 10.1093/eurheartj/ehu364 25265973

[18] Ruilope LM (2013). Long-term adherence to therapy: the clue to prevent hypertension consequences. Eur Heart J.

[19] Godin G (1989). The effectiveness of interventions in modifying behavioral risk factors in individuals with coronary heart disease. J Cardiopulmonary Rehab.

[20] Sheeran P (2002). Intention-behavioral relations: a conceptual and empirical review. Eur Rev Soc Psychol.

[21] WHO. Luxembourg: HiT in Brief. The Government of the Grand-duchy of Luxembourg. Ministry of Health. World Health Organization 2015 (acting as the host organization for, and secretariat of, the European Observatory on Health Systems and Policies). http://www.sante.public.lu/fr/publications/h/health-systems-transition-lux-2015/index.html.

[22] Tchicaya A, Lorentz N, Demarest S, Beissel J. Persistence of socioeconomic inequalities in the knowledge of cardiovascular risk factors five years after coronary angiography. Eur J Cardiovasc Nurs. 2017:1474515117720789. doi:10.1177/1474515117720789. 10.1177/1474515117720789 PMC5802545 28696137

[23] Einvik G, Ekeberg O, Sandvik L, Hjerkinn EM, Klemsdal TO (2009). Physical distress is associated with cardiovascular events in a high risk population of elderly men. BMC Cardiovasc Disord.

[24] Schimmack U, Oishi S (2005). The influence of chronically and temporarily accessible information on life satisfaction judgments. J Pers Soc Psychology.

[25] McDonnell LA, Riley DL, Blanchard CM, Reid RD, Pipe AL, Morrin LI, Beaton LJ, Papadakis S, Slovinec D’Angelo ME (2011). Gender differences in satisfaction with life in patients with coronary heart disease: physical activity as a possible mediating factor. J Behav Med.

[26] Cole JA, Smith SM, Hart N, Cupples ME. Systematic review of the effect of diet and exercise lifestyle interventions in the secondary prevention of coronary heart disease. Cardiol Res Pract. 2011;2011:232351. 10.4061/2011/232351 PMC3010651 21197445

[27] Godin G, Conner M, Sheeran P (2005). Bridging the intention-behaviour 'gap': the role of moral norm. Br J Soc Psychol.

[28] Crichton GE, Alkerwi A (2014). Association of sedentary behavior time with ideal cardiovascular health: the ORISCAV-LUX study. PLoS One.

[29] Eyre H, Kahn R, Robertson RM, Clark NG, Doyle C, Hong Y, Gansler T, Glynn T, Smith RA, Taubert K, Thun MJ; American Cancer Society; American Diabetes Association; American Heart Association. Preventing cancer, cardiovascular disease, and diabetes: a common agenda for the American Cancer Society, the American Diabetes Association, and the American Heart Association. Circulation*.* 2004; 29; 109(25):3244-3255. 10.1161/01.CIR.0000133321.00456.00 15198946

[30] Suls J, Bunde J (2005). Anger, anxiety, and depression as risk factors for cardiovascular disease: the problems and implications of overlapping affective dispositions. Psychol Bull.

[31] Smith L, Fisher A, Hamer M (2015). Television viewing time and risk of incident obesity and central obesity: the English longitudinal study of ageing. BMC Obesity.

[32] O'Neil A, Hawkes AL, Atherton JJ, Patrao TA, Sanderson K, Wolfe R, Taylor CB, Oldenburg B (2014). Telephone-delivered health coaching improves anxiety outcomes after myocardial infarction: the 'ProActive Heart' trial. Eur J Prev Cardiol.

[33] Godin G, Conner M (2008). Intention-behavior relationship based on epidemiologic indices: an application to physical activity. Am J Health Promot.

[34] Davidson KW, Mostofsky E, Whang W (2010). Don’t worry, be happy: positive affect and reduced 10-year incident coronary heart disease: the Canadian Nova Scotia health survey. Eur Heart J.

[35] Lloyd-Jones DM, Hong Y, Labarthe D (2010). Defining and setting national goals for cardiovascular health promotion and disease reduction: the American Heart Association’s strategic impact goal through 2020 and beyond. Circulation.

[36] Moriel G, Roscani MG, Matsubara LS, Cerqueira AT, Matsubara BB (2010). Quality of life in patients with severe and stable coronary atherosclerotic disease. Arq Bras Cardiol.

[37] Devaux M, Sassi F (2013). Social inequalities in obesity and overweight in 11 OECD countries. Eur J Pub Health.

[38] Alkerwi A, Sauvageot N, Donneau AF, Lair ML, Couffignal S, Beissel J, Delagardelle C, Wagener Y, Albert A, Guillaume M (2010). First nationwide survey on cardiovascular risk factors in grand-duchy of Luxembourg (ORISCAV-LUX). BMC Public Health.

[39] Môller L (2007). Gender differences in cardiovascular disease and comorbid depression. Dialogues Clin Neurosci.

[40] Hardcastle SJ, Taylor AH, Bailey MP, Harley RA, Hagger MS (2013). Effectiveness of a motivational interviewing intervention on weight loss, physical activity and cardiovascular disease risk factors: a randomised controlled trial with a 12-month post-intervention follow-up. Int J Behav Nutr Phys Act.

[41] Baumann M, Tchicaya A, Lorentz N, Le Bihan E (2016). Impact of patients’ communication with the medical practitioners, on their adherence declared to preventive behaviours, five years after a coronary angiography, in Luxembourg. PLoS One.

[42] Jakobsson S, Irewall AL, Bjorklund F, Mooe T (2015). Cardiovascular secondary prevention in high-risk patients: a randomized controlled trial sub-study. BMC Cardiovasc Disord.

[43] Viswanathan M, Golin CE, Jones CD, Ashok M, Blalock SJ, Wines RCM, Coker-Schwimmer EJL, Rosen DL, Sista P, Lohr KN (2012). Interventions to improve adherence to self administered medications for chronic diseases in United States. Ann Intern Med.

[44] Mummah S, King AC, Gardner CD, Sutton S (2016). Iterative development of Vegethon: a theory-based mobile app intervention to increase vegetable consumption. Int J Behav Nutr Phys Act.

[45] Pomey MP, Flora L, Karazivan P, Dumez V, Lebel P, Vanier MC, Débarges B, Clavel N, Jouet E (2015). Le « *Montreal Model* » : enjeux du partenariat relationnel entre patients et professionnels de la santé. Santé Publique.

